# Quality and best practice in medical laboratories: specific requests for autoimmunity testing

**DOI:** 10.1186/s13317-020-00134-0

**Published:** 2020-09-03

**Authors:** Ulrich Sack, Xavier Bossuyt, Hristina Andreeva, Péter Antal-Szalmás, Nicola Bizzaro, Dimitrios Bogdanos, Elena Borzova, Karsten Conrad, Marie-Agnes Dragon-Durey, Catharina Eriksson, Katarzyna Fischer, Anna-Maija Haapala, Ingmar Heijnen, Manfred Herold, Werner Klotz, Ana Kozmar, Andrea Tesija Kuna, Marcos López Hoyos, Vladimir A. Malkov, Lucile Musset, Eszter Nagy, Johan Rönnelid, Yehuda Shoenfeld, Tatjana Sundic, Alexandra Tsirogianni, Raivo Uibo, Maria José Rego Sousa, Jan Damoiseaux, on behalf of the European Autoimmunity Standardisation Initiative

**Affiliations:** 1 Medical Faculty, Institute of Clinical Immunology, University Leipzig, Leipzig, Germany; 2 Clinical and Diagnostic Immunology, KU Leuven, Louvain, Belgium; 3 Division of Immunology and Transfusion Medicine, Department of Laboratory Medicine, University Hospital of North Norway, Tromsoe, Norway; 4 Department of Laboratory Medicine, Faculty of Medicine, University of Debrecen, Debrecen, Hungary; 5 Laboratorio di Patologia Clinica, Ospedale San Antonio (Tolmezzo), Azienda Sanitaria Universitaria Integrata, Udine, Italy; 6 Department of Rheumatology and Clinical Immunology, Faculty of Medicine, University of Thessaly, Larissa, Greece; 7 Department of Dermatology and Venereology, I.M. Sechenov First Moscow State Medical University, Moscow, Russia; 8 Institut für Immunologie, Medizinische Fakultät “Carl Gustav Carus” der Technischen Universität Dresden, Dresden, Germany; 9 Department of Immunology, Georges Pompidou European Hospital, University of Paris, Paris, France; 10 Department of Clinical Microbiology/Clinical Immunology, Umeå University, Umeå, Sweden; 11 Individual Laboratory for Rheumatologic Diagnostics, Pomeranian Medical University in Szczecin, Szczecin, Poland; 12 Fimlab Laboratories, Tampere, Finland; 13 Division of Medical Immunology, Laboratory Medicine, University Hospital Basel, Basel, Switzerland; 14 Department of Internal Medicine II, Medical University of Innsbruck, Innsbruck, Austria; 15 Department of Laboratory Diagnostics, University Hospital Centre Zagreb, Zagreb, Croatia; 16 Department of Clinical Chemistry, University Hospital Center Sestre Milosrdnice, Zagreb, Croatia; 17 Servicio de Inmunología, Hospital Universitario Marqués de Valdecilla-IDIVAL, Santander, Spain; 18 Tafi Diagnostica, Vladivostok, Russia; 19 Department of Immunology, Assistance Publique-Hôpitaux de Paris, Groupe Hospitalier Pitié-Salpêtrière, Paris, France; 20 National Institute of Rheumatology and Physiotherapy, Budapest, Hungary; 21 Department of Immunology, Genetics and Pathology, Uppsala University, Uppsala, Sweden; 22 Zabludowicz Center for Autoimmune Diseases, Sheba Medical Center, Tel-Hashomer, Israel; 23 Department of Immunology and Transfusion Medicine, Stavanger University Hospital, Stavanger, Norway; 24 Medical Biopathologist, Immunology-Histocompatibility Department, “Evangelismos” General Hospital of Athens, Athens, Greece; 25 Department of Immunology, Institute of Bio- and Translational Medicine, University of Tartu, Tartu, Estonia; 26 Laboratório de Imunopatologia e Autoimunidade, UC Medicina Laboratorial, Grupo Germano de Sousa, Lisbon, Portugal; 27 Medical Immunology, Maastricht UMC, Maastricht, The Netherlands

**Keywords:** Autoimmunity, Autoantibodies, Medical laboratory, Quality, Accreditation

## Abstract

Special conditions associated with laboratory autoimmune testing are not well compatible with recent developments in regulatory frameworks such as EN/ISO 15189 accreditation or in vitro diagnostic medical device regulation (IVD-R). In addition, international recommendations, guidelines and disease criteria are poorly defined with respect to requirements on autoantibody testing. Laboratory specialists from Austria, Belgium, Croatia, Estonia, Finland, France, Germany, Greece, Hungary, Italy, Norway, Poland, Portugal, South Africa, Spain, Sweden, Switzerland, and The Netherlands collected information, reported national experience, and identified quality issues in relation to autoantibody testing that require consensus on interpretation of the regulatory frameworks and guidelines. This process has been organized by the European Autoimmunity Standardisation Initiative (EASI). By identifying the critical items and looking for a consensus, our objective was to define a framework for, in particular, EN/ISO accreditation purposes. Here, we present a review of current publications and guidelines in this field to unify national guidelines and deliver in this way a European handout on quality control and accreditation for laboratories involved in autoantibody testing. We focus on quality items that can be checked during accreditation visits. Despite various local varieties, we encountered an overwhelming dedication to quality assurance in all contributing countries.

## Introduction

Quality management is an important task for medical laboratories involved in patient care and translational research. It guarantees correct and meaningful laboratory results supporting medical care and innovation. New in vitro diagnostic regulations (IVD-R) in the European Union [1], and comparable regulations worldwide, define high standards for diagnostic test kits. If available, medical laboratories are obliged to use test kits that have been registered by the authorities. This market authorization will be granted based on relevant main characteristics, including value for the patients, by the providing manufacturers. The IVD-R, however, may be in conflict with the economic scenario in some countries, or even specific regions of a given country. If in such situations the local jurisdiction prevents the use of registered test kits, homemade assays may represent an alternative if extensively validated and secured in time by appropriate internal and external quality control.

For autoantibody testing, there are additional challenges to meet adequate quality management. These specific features of autoantibody testing require state-of-the-art solutions and include, but are not limited to:

- A huge variety and ever-increasing number of distinct autoantibodies with highly varying sample sizes to process.
- A common need for adding additional tests to be included in testing algorithms or to be subsequently ordered by the clinical specialist depending on the outcome of the primary testing.
- A combination of manual and automated testing.
- The need for different autoantibody test procedures depending on the requesting clinical partner (GP, clinical specialist, or other laboratories).

Accreditation according to EN/ISO 15189:2012 [2] defines general rules but is not the only framework for good laboratory quality. Several guidelines by medical boards, position papers by scientific medical organizations, international working groups for standardization, good scientific and laboratory practice, and various high-quality publications add up to define a framework how to make valid autoimmune diagnostics. This implies that independent of international and regional specifications, laboratory diagnostics should follow the same overarching rules irrespective the location or type of laboratory [3]. However, details in medical practice vary from country to country and may even differ within countries [4, 5]. Main factors that influence autoantibody requesting and subsequent testing are the prevalence of autoimmune diseases, distinctive diagnostic procedures between public and private sector, and balance between GPs, specialists and hospital centers, causing finally a highly different pre-test probability of autoimmune diseases. In addition, national regulatory procedures, laboratory’s financial constraints, and reimbursement policies, may hamper harmonization of autoimmune testing.

In this manuscript we identify and discuss critical items of analytical and clinical quality in autoimmune laboratory testing in relation to existing guidelines. We focus on commercially available assays. Although we realize that the development of in-house assays often precedes widespread introduction of tests for new autoantibodies into clinical practice, inclusion of in-house assays would further complicate the discussion. By defining the critical items and looking for consensus, our objective is to define a framework for EN/ISO accreditation purposes. We consider this work complementary to EULAR recommendations (https://ard.bmj.com/pages/collections/eular_papers/) that are merely established for clinical practice, not for the diagnostic methods and procedures in routine laboratories that we focus on.

We elaborate on the example of anti-nuclear antibodies (ANA, more appropriately defined as anti-cellular antibodies) and the related follow-up testing used to identify the antigen-specificity, i.e., antibodies to dsDNA, to extractable nuclear antigens (ENA), as well as to other intracellular components. There is now a wide agreement that the term ANA is outdated. Thus during the 2019 ICAP meeting in Dresden it was proposed to use HEp-2 indirect immunofluorescent assay (IIFA) as a replacement nomenclature for the test [6]. However, since there are various methods of detection and no consensus has been achieved, we continue to use the old nomenclature.

The ANA tests are probably the most challenging examples because the autoantibodies are related to a diversity of autoimmune diseases [6]. Multiple technologies are available with the explosion of the new chemiluminescence and multiplex automated systems, and tests lack harmonization. This has led to ongoing discussions on the most suitable testing algorithm fitted with the clinical approaches that are to be chosen, especially in the field of idiopathic inflammatory myopathies (IIM) or interstitial lung disease. In comparison, testing of ANCA for diagnosis of small vessel vasculitis is now based on clear recommendations [7], and parameters such as rheumatoid factors (RF) [8] or anti-citrullinated protein antibodies (ACPA) [9, 10] for rheumatoid arthritis diagnostics even moved to clinical core analyzers. However, the expanding field of new disease-related autoantibodies raises several additional challenges.

We analyzed current publications and guidelines in this field to unify national guidelines and deliver in this way a European recommendation on quality control and accreditation for laboratories involved in autoantibody testing. We focus on quality items that can be checked during accreditation visits.

### Current situation

Today, most European medical laboratories must undergo EN/ISO 15189:2012 [2] accreditation and in most countries, this has been implemented or is ongoing. The European cooperation for Accreditation (EA) is the roof organization of the national accreditation bodies. One of the central targets of EA is to harmonize the requirements laboratories must meet when applying for and maintaining EN/ISO 15189:2012 [2] accreditation. Meeting these requirements is less of a challenge in Clinical Chemistry but is a topic of interpretation for specialized laboratories. For the accreditation of specialized laboratories, such as in the field of immunology, accreditation bodies need scientific advice how to interpret the EN/ISO 15189:2012 [2]. Furthermore, because some countries do not have enough specialists in autoimmune testing or even in immune diagnostics, assessments are done by auditors without specific experience in this field.

There are some international guidelines including, at least in part, autoimmune testing. They have been developed by expert panels, represent state of the art diagnostics, and influence national practice. Most guidelines deal with the testing algorithm and give rather clinical advice. Some of the most relevant ones with respect to ANA have been published by IFCC [11], CLSI [12], ACR [13], EULAR [14], EASI/IUIS [15], and ICAP [16–18]. Though authoritative expert panels issued these guidelines, there are distinctions amongst the provided recommendations, leaving room for interpretation. As a result of this, national documents were derived in most countries (see also [15]). They have been published as guidelines, recommendations, or checklists. These various national rules have a lot in common. We collected information from Austria, Belgium, Croatia, Estonia, Finland, France, Germany, Greece, Hungary, Israel, Italy, Norway, Poland, Portugal, Russia, South Africa, Spain, Sweden, Switzerland, The Netherlands, and Ukraine. In most countries there are no specific rules for autoimmune testing in accredited laboratories and use of international recommendations is common, but not specified. This is true for Finland, France, Israel, Norway, Poland, Portugal, Russia, Sweden, and Ukraine. Belgium [19], Germany [20–24], Italy [25, 26], The Netherlands (https://medischeimmunologie.nl/cml/richtlijnen) and Switzerland (https://ssai.ch/organisation/commissions/cld/) have one or more national guidelines or recommendations for autoantibody testing. In Austria [27], France [28–30], Italy (http://www.gruppofirma.com/eng/), and Spain [31] there are publications by the national EASI groups (https://www.gfid-ev.de/easi/), but most of these documents have no formal regulatory role.

### Staffing and competency

Autoantibody testing must be performed by authorized and trained staff. EN/ISO 15189 accreditation not only requires determining the level of competence but also to assess and reassess the competence of each person. This can be done by pairwise examining EQA samples (“consensus training”) or common and rare samples under supervision and should be done and documented half-yearly. There are some further challenges:

- How to define the minimal size of the staff (and replacement)?
  The required number of staff members depends on technical and organizational aspects. There is a difference between automated and manual analyses, where the staff for manual analyses must be large enough to guarantee availability of skilled personnel during sick leaves, holidays, and unforeseen events. At the same time, it must be small enough to keep the competence updated in that selected group of individuals. Many laboratories have implemented highly complex workflows. The more decisions must be made by the staff, the more experience is required, and the more workload can be expected. There is a clear relationship between complexity of the assays (e.g., reading of IIFA) and the clinical interpretation (e.g., neurological antibodies) on the one hand and the number of staff required on the other.
  As a consensus it can be advised that a minimum number of laboratory employees (e.g., 3) trained in autoimmune testing according to specific national knowledge, skills and competence requirements (medical laboratory technicians, bioengineers, medical scientists, or European Specialists in Laboratory Medicine EuSpLM, recognized by the EFLM) must be in place. Autoimmune testing must be supervised by a specialist in Laboratory Medicine qualified according to regional or national legislation. They may need to be supported by medical advisors. Depending on workload, these may be part-time positions. Technicians workload might be divided with general immunology, allergology, microbiology, or other sections.
  As a rule of thumb, every laboratory technician should run once/weekly the automated platforms. The advising medical doctor in turn should be engaged with autoimmune testing once per week. Competence is not only analytical competence but also appropriate interpretation in the clinical context of the patient. In many countries, CME/CPD programs have been established and must also cover autoantibody testing. For example, German rules recommend about 4 internal trainings per year and one external specific training every second year to maintain the competency of the team, but this is not compulsory. In general, training requirements should be defined and should include internal and external training (differentially defined for technicians, staff and replacement of staff).
- Is this different for manual and automated assays?
  The laboratory processes of autoimmune testing must be supervised by experienced specialists even if performed in a fully automated setting.

### Laboratory space

There is increasing automation of autoantibody testing, including binding assays, testing by IIFA, and line/dot blots. Space requirements for automated instruments are not different from Clinical Chemistry or infectious serology. Nevertheless, manual processing of microscope slides, blots, ELISAs and even RIAs is still common practice in many laboratories and requires specific conditions and minimal space. These manual assays need a dedicated space for sample preparation and often require an adequate microscopy cabinet that can be dimmed.

### Equipment

If possible, high-throughput tests should be performed on fully automated systems that are linked to the laboratory information system. This avoids errors and gives more time for performing specialty tests and confirmation steps. The proper maintenance of these automated systems is crucial too. Fluorescence microscopes should be equipped with a LED light source to reduce maintenance efforts. If reading is not automated, data should be entered directly into laboratory systems; a middleware is the most common solution applied by laboratories today.

### Pre-analysis

A correct test request that is based on a pre-test clinical suspicion is critical to ensure optimal value of a test result. Request forms must be suitable to provide proper clinical information. Direct interaction with clinicians is difficult for commercial laboratories, but the requesting clinician should be encouraged to formulate clinical questions of relevance to differential diagnosis and management and to give tentative clinical diagnosis. In some settings, a specific form is implemented for antibody requests, providing clinical information to help interpreting test results.

Pre-analytical requirements for autoantibody detection are not time-critical, but quality should be as good as specified by manufacturer (icterus, lipemia, hemolysis, or other contamination; no repeated freeze-thawing). The sample quality also depends on patients’ status (prolongation of coagulation time in certain clinical situations; pregnancy etc.).

Most autoantibodies are detected in serum and are validated by the companies only for this matrix. Important exception is a series of neurological antibodies that may also be detected in cerebrospinal fluid. From laboratory processes perspective, plasma is favored since the lower centrifugation time and many manufacturers pointed out in their protocols that plasma (lithium heparin? citrate? EDTA?) can be used. If other body fluids are used, this will in general be outside the scope of the assay (unless the insert states otherwise).

### Testing strategies

**Fig. 1:**
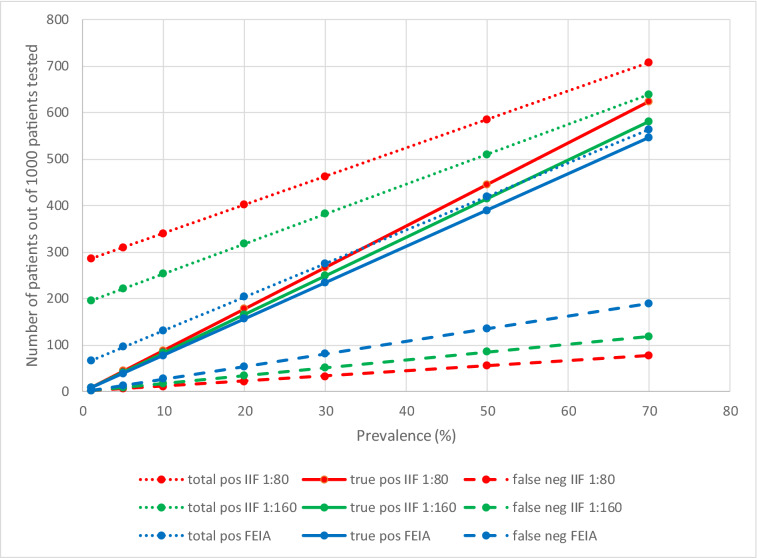
Comparison of ANA detection by IIFA with cutoff 1:80 or 1:160 versus screening for connective tissue disease-specific antibodies by EliA CTD (Thermofisher) screen. The figure shows the results of a simulation of the total number of positive test results as well as the true positive and false negative test results as a function of the prevalence (of connective tissue disease) for three different approaches/methods (screening for ANA by IIFA with cutoff 1:80 and cutoff 1:160 and screening for connective tissue-specific antibodies by EliA CTD Screen). The simulation is based on testing 1000 patients and the performance characteristics are from a recent meta-analysis [33]

The correct screening strategy for autoantibodies depends, at least in part, on the pre-test probability of the patient population. This varies by the requesting clinical discipline with different prevalence of positive test results [32, 33]. Furthermore, the number of positive findings also depends on the test characteristics (more positives for highly sensitive but less specific assays). In case of ANA, the prevalence of positive test results is < 30% [34]. For patients with a relatively high pre-test probability for SARD (requested by rheumatologists or clinical immunologists) or patients for which a SARD is to be excluded, the sensitive ANA IIFA may be the most adequate screening test. On the other hand, patients with a relatively low pre-test probability for SARD (requested by general practitioners or non-rheumatology/non-clinical immunology specialists) or for patients suspected of anti-SSA/Ro related diseases or IIM, alternative assays may be more appropriate [35, 36]. Finally, a patient may present with clinical manifestations completely in line with a specified diagnosis; testing in such patient is rather redundant unless the patient is to be included in a study or if—in rare situations—the test must be performed at presentation and during follow up to access response to treatment. Analytical sensitivity and specificity of IIFA ANA tests and binding assays are well known and result in different performances (Fig. 1). A special situation is the detection of antibodies that give information on prognosis. For example, some MSAs may not be useful for diagnosis, but they may give insight into prognosis (e.g., TIF-1gamma) [37–39]. This illustrates that clinical information is of utmost importance in order to perform tailor-made diagnostics. The alternative is to perform a combination of tests. There is evidence that combining IIFA with solid phase assays gives the most clinically useful information requested by general practitioners or non-rheumatology/non-clinical immunology specialists [36, 40–42].

Although there may be good arguments favoring immunoassays with defined antigens for screening instead of IIFA, there still are strong arguments for initial IIFA: reagents are generally cheaper, well-established systems for automation are available, the number of target antigens presented is higher and not all of them are available in commercially available solid phase assays (some may not even have been characterized), and several recommendations and disease criteria request IIFA.

### Validation of tests

Clinical validation must be distinguished from analytical validation. According to the new IVD regulations, clinical validation (i.e. diagnostic sensitivity and specificity) is the responsibility of the company. This information must be shared with the customers.

Analytical validation can occur at a multicenter level and can be supported by existing literature, but local verification must be performed as well. In this respect, laboratories sometimes have not enough positive samples. For a certain period, this can be overcome by proficiency test samples and control material, but the verification process must be finalized with real samples within around half a year. It may otherwise be questionable if these laboratories should perform the assay.

The choice of the analytics should primarily be based on the test characteristics and independent from methodology. Before implementing IVD tests, laboratories must verify them. The following test characteristics should be considered in such a verification:

- LoD (verification of starting dilutions for IIFA; verification of manufacturers’ cut-offs; published reference limits): the vast majority of tests are semi-quantitative CE-IVD and come along with a proposed decision limit.
- Sensitivity/specificity: Number of samples depends on the clinical indication and it is almost impossible to be achieved in a single laboratory. To start with a new assay for common autoantibodies it is worth to use 50 known positive and 100 known negative sera, although only a minimum of 30 comparisons with minimum of 10 samples per category, i.e. positive and negative, is required for kappa statistic [43].
- Precision requirements (intra-assay and inter-assay variation): For qualitative tests intra- and inter-assay variation cannot be calculated, but 10 replicates of positive and 10 replicates of negative samples should be analyzed in a couple of consecutive days in order to establish consistency of results. For semi-quantitative tests, intra- and inter-assay variation are to be calculated out of 10 intra-run replicates and 10 inter-run replicates, respectively, preferentially for 3 different levels: low positive, intermediate positive and high positive (Senant M et al., submitted). For autoantibody tests, a CV of 20% or 1 titer-step are well accepted as target CV; CV of negative samples should not be calculated because it can be very high without any relevance.
- Further parameters such as linearity and measuring range, Hook-effect or prozoning, and general handling issues like robustness or carry-over may be verified but are less relevant when applying CE-IVDs. The same is true for interferences (see pre-analytical requirements).

For analytical validation, qualitative and quantitative assays require different approaches. Until today, there are no true quantitative tests for autoantibody detection. Because such tests detect a polyclonal mixture of antibodies, even well-standardized parameters such as RF are not quantitative; there is no reference method. This also means that results are test kit-specific. It is possible to perform the analytical verification semi-quantitatively and define discrete classes (negative, intermediate, positive).

The challenges here include (but are not limited to):

- The number of samples available for rare antibodies: Most laboratories use a 5 × 5 approach (each sample is analyzed five times in parallel on five occasions). Missing positive samples must be supplemented by positive controls and/or EQA samples. This is a problem since some antibodies are also rarely positive in EQA samples (Jo-1 or ribosomal P, or antibodies in paraneoplastic syndromes, for example). It can be considered to make an additional (artificial) positive sample by diluting the highly positive one with negative sera, because each lot has to be approved;
- How to deal with multiplex assays? Basically, all entities that are reported to the clinic must be verified in the laboratory;
- How to deal with linearity? Autoantibodies are a mixture of low–medium–high affinity and are not always linear upon dilution.

The Dutch initiative for national validation/verification of autoantibody assays gives clear advice [44]. A common and not yet solved challenge is the verification and quality control in multiplex assays. The typical procedure assumes that the testing of exemplary antigens is enough.

### Analyzing indirect immunofluorescence assays

Slide reading should ideally be done with direct importing of results into a laboratory information system through middle ware or must be double-checked. Pattern recognition software is not (yet) fully reliable and can only replace one of two mandatory readers if at all.

### Analyzing dot-/line-blots

Dot-blots and line-blots are not really blotted but are rather conventional immunoassays. They are intended to analyze autoantibodies to multiple antigens in parallel. For quantification, control lanes and internal standards can be part of the test kit. Some manufacturers only provide a control lane showing IgG content in the strip; thus, there are no graded internal controls for the individual autoantibodies analyzed in the test system. In case of disease specific antibody panels, i.e. screening for myositis/scleroderma-typical autoantibodies, these assays are increasingly used, but verification of the rare specificities is often lacking.

### Analyzing binding assays

Immunological binding assays for ANA, ENA, ANCA, RF or ACPA are available as microtiter plates or as test kits for common analyzers in Clinical Chemistry. While microtiter plates are processed as a batch with calibrators and controls per plate, random access analyzers follow the common quality rules in Clinical Chemistry (lot-specific master curves).

### Internal quality control

Diagnostic laboratories are bound to kit-intern quality controls of CE-labeled IVD. Replacing a kit-intern control means modifying the test and requires validation efforts. Additional third-party controls are highly recommended. One important reason to have in-house internal controls in the lab is the longevity of stored properly. In-house internal controls might last a decade and will still be the same when the companies change their kit controls in an unpredictable way. In-house controls based on positive findings can be applied but must be profoundly characterized and well documented. Internal control level should be in the measuring range, preferentially near the cut-off.

For solid phase assays and IIFA, internal controls must cover as many antigens as possible. For IIFA, control sera can also be applied as titer-control to proof stability of reagents, slides, microscope, and any other component influencing microscopic imaging.

Lot approval should be done with the use of confirmed samples with known laboratory results. If possible, clinical information about these samples should be known to the laboratory specialist.

Results of internal controls should be transferred into the quality control charts of the laboratory and managed accordingly.

### External quality assessment

In some countries, there is a difference between external quality assessment (EQA), which is performed on a voluntary basis, and proficiency testing, which is obligatory and involves restrictive measures. Participation in proficiency testing is mandatory for medical laboratories. They should cover the full scope of parameters offered by the laboratory within a defined time period. For some kinds of specific test kits this is a challenge: myositis blots, neuronal blots etc. This often means that proficiency testing providers from other countries must also be involved. A good database for EQA providers can be found here: https://www.eptis.bam.de/eptis/WebSearch/main. For rare antibodies there is often no EQA. Sample exchange with other laboratories can assist efforts to overcome this issue. Clinically validated specimens should be used but must be defined thoroughly. The rules for such exchanges must be defined in advance and should consider various test systems if possible.

### Turn-around time

Turn-around time depends on the setting. In a hospital, a preliminary result of ANA/ENA analysis should be available within 48 to 72 h, but this can result in need for correction by unforeseen results of additional follow-up tests. ANCA or GBM are more urgent than other autoantibodies and must be reported within 24 h if requested as such; therefore, rapid test performance must be guaranteed.

For out-patients, there is in general more time to finalize everything until the follow-up consultation. To remain within an acceptable timeframe, rare tests can be outsourced.

## Result reporting

Reporting autoantibody results needs good knowledge of test characteristics in relation to the clinical manifestations of the patient. Generalized interpretative comments on autoantibody test results are important at least, when autoantibodies with a significant clinical correlation are found [45]. It is not uncommon to get controversial results when combining tests. Commenting on such true or apparent discrepancies may help result interpretation by the recipients. Interpretation of results by an inexperienced physician is also an evolving issue (especially for ANA positive tests with a cytoplasmic pattern or rare autoantibodies). Performing an additional test in such situations is not very helpful without feedback from the requester [45]. Obviously, interpretation of discrepant results is a challenge for the laboratory specialist.

In most laboratories, ANA IIFA results are reported in titers starting from 1:40 or in light-intensity units. Low ANA titers can be less specific and could be misleading, mostly 1:160 is considered the first, clinically relevant titer. High-titer sera should be diluted maximum down to 1:1280 [15]; further dilution will not add clinical value.

ANA reports should be done according to ICAP recommendations [16], at least using the nomenclature. Depending on the clinical pre-test probability, there are good arguments to report specific likelihood ratios [46, 47] in addition to titers.

Narrative or customized comments should be implemented in the report. This can also include the test kit applied.

## Conclusion

We hope that the hints provided here help to overcome inadequate requests of rules that cannot be fulfilled by laboratories involved in autoimmune testing. We have presented the current situation and tried to propose unified requirements for laboratories undergoing and maintaining accreditation according to EN/ISO 15189:2012 [2]. These requirements also refer to EN/ISO 17025 compliant laboratories and other frameworks (Guidelines of the German Medical Association [48], GMP QC, notified bodies, and others more). We are sure this is beneficial for all parties involved in accreditation: laboratories will know how to best prepare for assessments, assessors will know what to expect, and accreditation bodies know that EN/ISO 15189:2012 [2] has been implemented everywhere in the same way.

## Data Availability

Not applicable

## Abbreviations

ACPA: Anti-citrullinated protein antibodies
ACR: American College of Rheumatologists
ANA: Anti-nuclear antibodies
ANCA: Anti-neutrophil cytoplasmic antibodies
CE-IVD: European Union compliant in vitro diagnostic medical device
CME: Continuing medical education
CPD: Continuing professional development
CLSI: Clinical and Laboratory Standards Institute
CTD: Connective tissue disease
EA: European Accreditation
EASI: European Autoimmunity Standardisation Initiative
EFLM: European Federation of Laboratory Medicine
EN: European norm
ENA: (Antibodies against) extractable nuclear antigens
EQA: External quality assessment
EULAR: European League Against Rheumatism
EuSpLM: European Specialists in Laboratory Medicine
GBM: Glomerular basement membrane
GP: General practitioner
HEp-2: Human epithelial type 2
ICAP: International consensus on antinuclear antibody patterns
IFCC: International Federation of Clinical Chemistry
IIFA: Indirect immunofluorescence assay
IIM: Idiopathic inflammatory myopathies
ISO: International Standardization Organization
IUIS: International Union of Immunological Societies
IVD-R: In vitro diagnostic medical device regulation
LED: Light emitting diode
LoD: Limit of detection
MSA: Myositis-specific autoantibodies
POCT: Point of care testing
RF: Rheumatoid factor
SARD: Systemic autoimmune rheumatic diseases
TIF-1gamma: Transcriptional intermediary factor 1 gamma
